# A Cross-Sectional Study Exploring a Mediation Model of Nature Exposure and Quality of Life: The Roles of Nature-Based and Overall Physical Activity

**DOI:** 10.3390/bs15111442

**Published:** 2025-10-23

**Authors:** Migle Baceviciene, Rasa Jankauskiene

**Affiliations:** Faculty of Health Sciences, Health Research and Innovation Science Center, Klaipeda University, 92294 Klaipeda, Lithuania; rasa.jankauskiene@ku.lt

**Keywords:** nature, green exercise, physical activity, well-being, quality of life, equity

## Abstract

This cross-sectional study examined whether physical activity (PA) in nature and overall PA mediate the relationship between nature exposure and quality of life (QoL) across four domains: physical, psychological, social, and environmental, while controlling for perceived financial security. A cross-sectional online survey was conducted, involving 924 adults aged 18 to 79 years (m = 40.0, SD = 12.4); 73.6% were women. Nature exposure, PA in nature, overall PA, and financial security were assessed using nationally language-validated self-report scales and questionnaires. QoL was measured using the WHOQOL-BREF, covering four domains. Mediation models were tested using the regression-based PROCESS macro with 5000 bootstrapped samples. Nature exposure was positively associated with both types of PA and all QoL domains, while financial security was positively linked to PA in nature. PA in nature significantly mediated the relationship between nature exposure and psychological QoL, but not the other domains. In contrast, overall PA was a significant mediator across all QoL domains. In all models, nature exposure and financial security remained significant direct predictors of QoL. Bootstrapped confidence intervals confirmed the significance of indirect effects through overall PA for physical, psychological, social, and environmental QoL. While nature exposure was independently associated with better QoL, this relationship was partly explained by PA. These findings highlight the broader role of PA in linking nature exposure to QoL and underscore the importance of supporting active lifestyles in nature to enhance QoL. To achieve a higher QoL, policies that increase access to and opportunities for nature-based physical activity should be implemented.

## 1. Introduction

A substantial body of research has demonstrated that spending time in natural environments has a positive impact on human health and overall well-being (Coventry et al., 2021; Hartig et al., 2014; Martin et al., 2020; Nejade et al., 2022; Twohig-Bennett & Jones, 2018). Natural environments are typically defined as areas that exist in nature and have experienced minimal human alteration or intervention (Hartig et al., 2014). These spaces are characterized by functioning ecosystems where plants, animals, and other natural elements coexist with limited or no significant human disturbance. In urban areas, the natural environment encompasses parks, gardens, and a variety of flora and fauna found in cities. This also encompasses community gardens, green roofs, street trees, green verges, and bodies of water, collectively referred to as “green infrastructure” (Halecki et al., 2023). These natural features provide urban populations with significant environmental and health benefits (Zhang & Qian, 2024).

A comprehensive review of 103 observational and 40 interventional studies, encompassing approximately 100 health outcomes, found that exposure to nature significantly reduced diastolic blood pressure, salivary cortisol levels, heart rate, and the risk of diabetes, as well as both all-cause and cardiovascular mortality (Twohig-Bennett & Jones, 2018). Similarly, a systematic review of nature-based interventions—structured activities that promote engagement with natural settings—found these interventions to be effective in improving mood, reducing anxiety, enhancing positive affect, and lowering negative affect. However, the evidence for physical health benefits from such interventions was less conclusive (Coventry et al., 2021). A more recent review focusing on meta-analyses of the psychological and physical impacts of connection with nature found consistent evidence across diverse experimental studies. It concluded that physical engagement with natural environments supports improvements in cognition, social skills, physical and mental health, and fosters a deeper psychological connection to nature (Barragan-Jason et al., 2022). Beyond individual psychological and physical benefits, green spaces also offer significant social, economic, and environmental advantages. These include enhanced social interaction, increased property values, ecosystem services, and reduced pollution—all of which contribute to improved health, greater social and material well-being, and an overall higher quality of life (Mensah et al., 2016). However, according to the international survey involving 14 European countries and 14,745 respondents, the relationship between humans and the natural world is deteriorating, contributing to climate change and the loss of biodiversity. Results of this study suggested that consumption and commerce had a moderately strong negative relationship with nature connectedness (M. Richardson et al., 2022).

Engaging in physical activity offers a valuable opportunity for individuals to connect with nature and benefit from its wide-ranging psychological and physical effects (Banwell et al., 2024; Martin et al., 2020). A recent systematic review concluded that there is a positive association between access to green spaces and physical activity (Ramezanghorbani et al., 2025). Physical activity in nature encompasses more than just intentional exercise. It includes a range of everyday behaviors such as working outdoors (e.g., gardening), active transportation (e.g., walking or cycling to work), and recreational pursuits (e.g., hiking or casual cycling) (Hartig et al., 2014). Research consistently shows that physical activity performed in natural environments provides greater physiological and psychological health benefits than physical activity in urban or indoor settings (Laezza et al., 2025; Lahart et al., 2019; Pretty et al., 2005; Wicks et al., 2022). In particular, people often find exercise in nature to be more restorative compared to similar activities in built environments.

According to Attention Restoration Theory (R. Kaplan & Kaplan, 1989), exposure to natural environments replenishes depleted attentional resources and reduces cognitive fatigue, thereby enhancing individuals’ capacity to engage in physical activity in general, not only in natural settings (R. Kaplan & Kaplan, 1989; S. Kaplan, 1995; Menardo et al., 2021). In contrast to urban environments, which often demand sustained and effortful attention, nature captures attention in a more effortless way, allowing directed attention systems to rest and recover. Stress Reduction Theory (Ulrich et al., 1991) provides a complementary perspective, suggesting that natural environments promote rapid affective and physiological recovery from stress, which may also be related to greater participation in physical activity (Stults-Kolehmainen & Sinha, 2014). Together, these perspectives offer a rationale for examining both nature-based and overall physical activity as potential mediators in the association between nature exposure and quality of life.

At the same time, restoration alone does not fully explain the connection between nature-based activity and well-being (Pasanen et al., 2018). Scholars have argued that natural settings provide unique behavioral opportunities that differ from those available in built or urban contexts. Realizing these nature-based behavioral opportunities requires perceptual and behavioral variability, which draws individuals to become physically, psychologically, and/or emotionally connected with the natural environment (Araújo et al., 2019). Supporting this, recent evidence indicates that exercising in outdoor natural areas is linked to better cognitive functioning and greater frontal brain thickness in adults (Baena-Extremera et al., 2024).

Physical activity is widely regarded as a pathway through which exposure to nature may influence health and quality of life (Hartig et al., 2014; Korpela et al., 2014). However, while a growing body of evidence suggests that exposure to nature enhances the benefits of physical activity, a key question that previous literature has barely addressed is how these benefits interact (Shanahan et al., 2016). Although there is increasing evidence of the mediating role of physical activity in the relationship between exposure to nature and health, the existing evidence remains inconclusive. Some studies found no mediating role for physical activity (de Vries et al., 2013; Maas et al., 2008; E. A. Richardson et al., 2013) while other studies have found evidence of its effects (van den Berg et al., 2019; Kruja et al., 2024; Liao et al., 2023; Murrin et al., 2023; Sugiyama et al., 2008). However, in previously mentioned studies, physical activity was assessed as a total rather than specifically as physical activity performed in natural settings. For instance, in the study by Sugiyama et al. (2008), it remains unclear whether health benefits were associated with walking to green spaces or within them (Sugiyama et al., 2008). Nevertheless, some studies addressed this distinction. One study found that leisure-time green physical activity, but not transport-related activity, mediated the relationship between perceived green space and mental well-being (Liao et al., 2023). Similarly, de Vries et al. (2013) reported that while total physical activity did not mediate the relationship between greenery and health, physical activity specifically within public green spaces did serve as a mediator (de Vries et al., 2013). Further evidence comes from an intervention study by Petrunoff et al. (2021), which demonstrated that park-based physical activity at three-month follow-up was a strong and consistent mediator for the observed effects of the intervention (park physical activity prescription) on psychological quality of life (Petrunoff et al., 2021). These findings highlight the importance of distinguishing between general physical activity and physical activity in nature when examining their potential contributions to health and well-being. Continued research is needed to clarify which forms of physical activity are most influential in enhancing health outcomes associated with nature exposure.

Overall physical activity includes all forms of bodily movement performed during leisure, work, household tasks, or transportation, regardless of the setting. It is defined as any movement produced by skeletal muscles that results in energy expenditure (Caspersen et al., 1985). Nature-based physical activity refers to physical activity carried out in environments dominated by natural features such as parks, forests, rocks, or lakes (Araújo et al., 2019). Although these two types of activity may overlap, they can lead to different psychological and physiological responses. While exercise benefits multiple bodily systems—including the immune, musculoskeletal, respiratory, and hormonal systems (Posadzki et al., 2020)—nature-based activity additionally provides restorative and emotional benefits associated with contact with natural stimuli (Christiana et al., 2021). Thus, both types of activity could serve as separate mediating pathways between nature exposure and quality of life.

However, less is known about whether total physical activity and nature-based physical activity mediate the relationship between nature exposure and various domains of well-being. While a growing number of studies have examined the mediating role of physical activity in the link between nature exposure and psychological well-being, much less attention has been given to its potential role in physical, social, and especially environmental well-being (Laezza et al., 2024). General physical activity is consistently linked to improvements in multiple domains of quality of life through well-documented physiological and psychosocial mechanisms. Regular engagement in physical activity enhances the physical domain by improving cardiovascular fitness, muscular strength, and overall vitality, thereby reducing fatigue and the risk of chronic disease (Posadzki et al., 2020). In the psychological domain, exercise contributes to mood regulation and stress reduction through neurobiological pathways involving endorphin release, improved sleep, and reduced cortisol levels (Childs & de Wit, 2014; Passos et al., 2014). The social domain is beneficial when physical activity occurs in group or community settings, as it fosters social interaction, support, and a sense of belonging (Eime et al., 2013).

However, nature-based physical activity might influence quality of life through mechanisms that extend beyond those of general physical activity. In addition to the physical benefits associated with exercise, exposure to natural environments contributes to psychological restoration, including reduction in mental fatigue, anxiety, and depressive symptoms, as proposed by attention restoration theory and stress reduction theory (Ulrich et al., 1991; R. Kaplan & Kaplan, 1989). The social domain may be strengthened through shared outdoor experiences that foster community bonding, cooperation, and feelings of connectedness to both people and place (Korpela et al., 2014). Importantly, nature-based activity also enhances the environmental domain, promoting greater environmental awareness, place attachment, and satisfaction with one’s surroundings (Teixeira et al., 2023). These combined effects suggest that nature-based physical activity supports quality of life through both the physiological pathways of exercise and the restorative and relational benefits of nature contact. Testing mediation effects across quality-of-life domains can help clarify the distinct and overlapping mechanisms through which nature exposure enhances well-being. Such insights can, in turn, inform the development of more inclusive and effective approaches to urban planning, green space design, and community-based health interventions. Ultimately, exploring these domains is essential for advancing public health science and guiding evidence-based policy (Mensah et al., 2016).

As previously noted, access to natural environments in residential areas has been consistently associated with higher levels of outdoor physical activity (Calogiuri & Chroni, 2014; Pyky et al., 2019). However, lower socioeconomic status might negatively influence participation in nature-based physical activity. While engaging in physical activity in natural settings is generally free of charge—making it a potentially accessible option for individuals from lower socioeconomic backgrounds—it does not guarantee equal access or use. People from disadvantaged backgrounds often live in neighborhoods that are located farther from green spaces (Schüle et al., 2019). Furthermore, such neighborhoods are frequently exposed to higher levels of air and noise pollution (European Environment Agency, 2018), which may further discourage outdoor activity. Therefore, understanding how socioeconomic status shapes the relationship between nature exposure, physical activity, and quality of life is crucial for addressing health and physical activity inequalities. It also highlights the need for inclusive urban planning and equitable access to natural environments to ensure that the health benefits of nature are available to all segments of the population.

**Figure 1:**
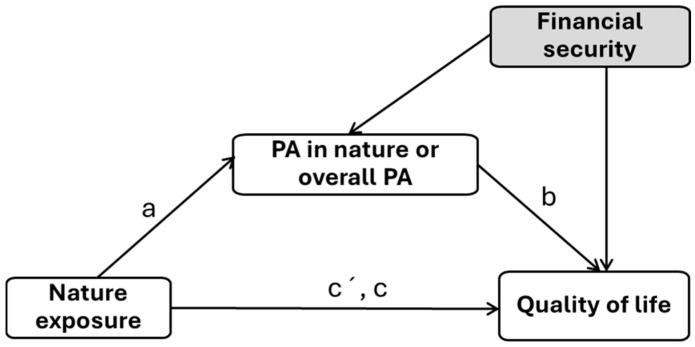
Theoretical model of the study, illustrating nature exposure as the independent variable, physical activity in nature or overall physical activity as the mediator, and quality-of-life domains as the dependent variables; financial security is included as a covariate to examine its effects on both the mediator and the dependent variables. Note. PA—physical activity. a—effect from the independent variable to the mediator; b—effect from the mediator on the outcome, c’; c—direct and indirect effects from the independent variable on the outcome.

The aim of the present study was to examine whether overall physical activity and physical activity in nature mediate the relationship between nature exposure and quality of life across four domains: physical, psychological, social, and environmental, while controlling for perceived financial security. We expected that nature exposure would be positively associated with both overall physical activity and physical activity in nature, as well as with overall quality of life. Furthermore, we hypothesized that overall physical activity and physical activity in nature would mediate the relationships between nature exposure and the various quality-of-life domains, after accounting for financial security (Figure 1).

## 2. Materials and Methods

### 2.1. Procedure

The study employed a cross-sectional survey design. Participants were recruited using a non-probability sampling approach. Inclusion criteria were set only for age (18 years and over) and language spoken (Lithuanian). Prior to participation, individuals were provided with information about the purpose of the study, the survey content, and the estimated time required to complete the questionnaire (approximately 25–30 min). To ensure data integrity, the survey platform was configured to accept only one response per IP address.

The survey was administered via Google Forms. All questions were set as mandatory, and responses were limited to predefined answer options, which helped eliminate missing values, invalid entries, and manual data entry errors. The anonymous survey link was distributed through sponsored advertisements on Facebook, targeting all major municipalities across the country.

Participants were required to provide digital informed consent before accessing the survey measures. Those who declined consent were acknowledged, and their participation was automatically terminated. Additionally, participants retained the option to withdraw at any time by closing the browser window. This study was approved by the university‘s Social Research Ethics Committee (Protocol No SMTEK-60) and implemented between January and April 2021. The present study is a part of a more extensive international study, Body Image and Nature Survey (BINS) (Swami et al., 2022).

### 2.2. Study Participants

The inclusion criteria were minimal: individuals had to be 18 years of age or older and fluent in Lithuanian. While nine participants refused to provide consent and participate, the final sample contained no missing data and consisted of 924 adults aged 18–79 years with a mean age of 40.0 ± 12.4 years. Of the total sample, 26.4% were men (n = 244), and the remaining 73.6% were women (n = 680).

### 2.3. Study Measures

The frequency of physical activity in nature was measured using a single item: “How often do you exercise, go for a walk, cycle, or engage in physically demanding activities in a natural environment (e.g., forest, park)?” Response options followed a 6-point frequency scale: 1 = never or very rarely, 2 = 2–3 times a month, 3 = once a week, 4 = 2–4 times a week, 5 = 5–6 times a week, and 6 = every day. This item was adapted from a Lithuanian national survey, with modifications to focus specifically on physical activity conducted in natural settings (Klumbiene et al., 2015). To reduce seasonal bias, participants were instructed to report the frequency of their physical activity in nature separately for spring, summer, autumn, and winter. An average physical activity in nature score across the four seasons was computed for analysis. The internal consistency in this study was excellent, with a Cronbach‘s α of 0.94.

To assess overall physical activity, the Godin Leisure-Time Exercise Questionnaire (LTEQ) was administered (Godin, 2011). Participants indicated the number of weekly sessions, each lasting at least 15 min; they engaged in light, moderate, and vigorous physical activity. In line with the questionnaire’s scoring protocol, responses were weighted by intensity: light activity sessions were multiplied by 3, moderate by 5, and vigorous by 9. These weighted values were then summed up to produce an overall leisure-time physical activity score for each season, representing weekly physical activity levels during warm season (late spring, summer, and early autumn) and cold and dark season (late autumn, winter, and early spring). For the analysis, summer and winter season scores were averaged.

Nature exposure was measured using the Nature Exposure Scale (NES) developed by Kamitsis and Francis (Kamitsis & Francis, 2013). This scale has been found to have a unidimensional structure and satisfactory internal consistency in prior research on the psychological effects of contact with nature, including its Lithuanian adaptation (Matukyniene et al., 2021). The NES comprises four items that capture individuals’ perceived exposure to natural environments, both in daily life and in less routine settings, as well as their awareness of natural surroundings. Responses are recorded on a 5-point Likert scale ranging from 1 (not at all) to 5 (very often), with higher mean scores reflecting greater reported engagement with nature. In the current study, the NES demonstrated acceptable internal reliability, with Cronbach’s α of 0.69.

Quality of life (QoL) was assessed using the World Health Organization Quality of Life–BREF (WHOQOL-BREF) questionnaire, a widely used 26-item instrument that measures perceived well-being across four domains: physical, psychological, social, and environmental quality of life (Skevington et al., 2004). WHOQOL-BREF domain scores were computed according to the official WHO guidelines: three items were reverse-coded, and the domain scores were calculated as the mean of corresponding items and then transformed to a 0–100 scale using the official SPSS Syntax provided by the World Health Organization. The tool has been validated in diverse populations and is appropriate for both clinical and non-clinical settings, and has demonstrated good psychometric characteristics in the Lithuanian sample (Ducinskiene et al., 2002). In the present study, the WHOQOL-BREF demonstrated good internal consistency, with Cronbach’s alpha values of 0.79 for the physical domain, 0.83 for psychological, 0.80 for social, and 0.80 for environmental quality of life.

Participants reported their gender and age in years. Educational attainment was assessed with five response options: secondary education, in full-time studies, undergraduate degree, postgraduate degree, or other. Family situation was measured using the following categories: single, single but in a committed relationship, married, or other. Place of residence was assessed with five options: capital, capital suburb, provincial city with more than 100,000 inhabitants, provincial city with more than 10,000 inhabitants, and rural area. Ethnicity was self-reported as belonging to the ethnic majority, ethnic minority, or unsure. Perceived financial security was assessed by asking participants to compare their situation to others, with three response options: less secure, equally secure, or more secure. Body mass index (BMI) was calculated from self-reported weight and height and categorized into four groups based on WHO guidelines: underweight (BMI < 18.5), normal weight (18.5–24.9), overweight (25.0–29.9), and obese (≥30.0) (World Health Organization, 2000).

### 2.4. Statistical Analysis

The sample size of 924 participants exceeds the minimum required to detect small-to-moderate effects in both correlation and mediation analyses with adequate statistical power. According to Cohen’s (2013) guidelines, detecting a small correlation (r = 0.20) at α = 0.05 and power = 0.80 requires a minimum of 193 participants (Cohen, 2013). For mediation analysis with one mediator and one covariate, a sample size of approximately 462 participants is sufficient to detect a small indirect effect using percentile bootstrap methods (Fritz & MacKinnon, 2007). Therefore, the current sample provides sufficient statistical power (>0.95) to detect small effects, minimize Type II error, and ensure reliable estimation of indirect effects in the proposed mediation models.

Descriptive statistics were computed to summarize sample characteristics, with frequencies and percentages reported for categorical sociodemographic variables. The distribution of continuous variables was assessed using skewness, with all variables falling within acceptable limits (Skewness < 1), indicating approximate normality. Internal consistency of the scales was assessed using Cronbach’s alpha (α). Alpha values ≥ 0.70 were interpreted as acceptable, values ≥ 0.80 as good, and values ≥ 0.90 as excellent (Vaske et al., 2017).

Based on this, Pearson’s correlation coefficients were used to examine associations between nature exposure, physical activity (in nature and overall), and quality of life (QoL) domains. For financial security, which was measured as an ordinal variable, Spearman’s rank-order correlation was used. Following Cohen’s guidelines, correlation effect sizes were interpreted as small (r = 0.10–0.29), medium (r = 0.30–0.49), and large (r ≥ 0.50) (Cohen, 1992).

To test the hypothesized mediation model, four models were conducted, treating physical activity in nature and overall physical activity as mediators between nature exposure (independent variable) and each QoL domain (dependent variables: physical, psychological, social, and environmental). While testing first for the moderating effects in all the analyzed paths, financial security was included as a covariate in all models to account for potential confounding effects. Financial security was treated as a continuous ordinal covariate, assuming a linear relationship between adjacent categories (1 = less secure, 2 = like most, 3 = more secure). Multicollinearity was not detected, with variance inflation factor (VIF) values ranging from 1.03 to 1.17. Standardized regression coefficients (β) were reported to describe and compare the importance of individual predictors, while determination coefficients (R^2^) were used to show the variance explained by the models. Unstandardized coefficients (B) and *p*-values were also reported for interpretability. The significance of the mediation pathway was evaluated using bootstrap resampling with 5000 samples, and an indirect effect was deemed significant if its 95% confidence interval did not include zero (Preacher & Hayes, 2008). The proportion of the total effect mediated (% mediated) was computed as the ratio of the indirect to the total effect (a × b)/c × 100, providing an estimate of how much of the association between nature exposure and quality of life operates through the mediator (Hayes, 2022). A significance threshold of *p* < 0.05 was applied. All analyses were conducted using software SPSS v.29 (IBM Corp., Armonk, NY, USA), while mediation models were tested with the SPSS PROCESS v. 4.2 by A. Hayes (Rockwood & Hayes, 2020).

## 3. Results

Table 1 presents sociodemographic characteristics of the study sample (mean age was 40.0 ± 12.4 years, range from 18 to 79). Women, married respondents with a university degree, inhabitants of provincial cities representing the ethnic majority, were dominant in this sample. Regarding financial security, 17.1% of participants reported feeling less secure than others, 64.8% felt equally secure, and 18.1% perceived themselves as more financially secure. The average body mass index (BMI) was 24.8 (SD = 4.6) kg/m^2^, with values ranging from 16.4 to 44.9 kg/m^2^. Based on the BMI classification, 3.8% of the sample were underweight, 55.7% had normal weight, 28.3% were overweight, and 12.2% fell into the obese category.

Table 2 presents the correlation coefficients among the study variables and means (SDs). Nature exposure was positively and significantly correlated with physical activity in nature and overall physical activity, suggesting that individuals with higher nature exposure tend to engage more in both general and nature-based physical activity. Physical activity in nature showed a moderate positive correlation with overall physical activity, supporting their conceptual relatedness while maintaining enough distinction to treat them as separate constructs. Physical activity in nature and overall physical activity were also significantly correlated with all four quality of life (QoL) domains, though the strength of these associations was small to moderate. Financial security demonstrated significant positive correlations with all QoL domains as well as with both nature exposure, physical activity in nature, and overall physical activity, with the strongest correlation observed with environmental QoL. Overall, the correlation matrix supports the proposed relationships between nature exposure, physical activity, and QoL outcomes, and highlights financial security as a relevant covariate in these associations.

Table 3 presents the results of four mediation models examining the role of physical activity in nature in the relationship between nature exposure and QoL domains, with financial security included as a covariate (in preliminary analyses, no moderating effects of financial security were found on any paths). In all models, nature exposure was significantly associated with physical activity in nature (B = 0.65, β = 0.35, *p* < 0.001, path a), and this path was consistent across the analyses (this step is presented only in Model 1). Next, Model 1 demonstrates a significant effect of physical activity in nature on physical QoL. However, considering one path relatively weaker, the bootstrapped indirect effect between nature exposure and physical QoL is not significant, suggesting no mediating effect of physical activity in nature in the association between nature exposure and physical domain of QoL (B = 0.53, 95% BootCI = −0.02–1.09). Similarly, Model 2 showed a significant mediating effect of physical activity in nature for the psychological domain of QoL with a significant indirect effect (B = 0.71, 95% BootCI = 0.15–1.32). Lastly, Models 3 and 4 revealed non-significant associations between physical activity in nature and QoL in social and environmental domains, suggesting no mediating role of physical activity in nature (indirect effects, B = 0.47, 95% BootCI = −0.30–1.30 and B = 0.22, 95% BootCI = −0.28–0.74, respectively). Specifically, while path a (nature exposure → physical activity in nature) was consistently strong, path b (physical activity in nature → QoL) for physical, social, and environmental domains was weak or non-significant. This explains the absence of significant indirect effects in these models. However, nature exposure and financial security remained significant direct predictors of QoL in all four domains.

Next, to examine whether overall physical activity mediates the relationship between nature exposure and different QoL domains, four regression-based mediation models were tested, controlling for financial security (Table 4). In preliminary analyses, no moderating effects of financial security were found on any paths. Nature exposure was significantly associated with overall physical activity, a relationship consistent across all four models, forming the first step of each mediation analysis. In contrast, financial security was not a significant predictor of overall physical activity. Further, overall physical activity was positively associated with all four QoL outcomes: physical, psychological, social, and environmental. In each model, nature exposure also remained a significant direct predictor of QoL. Financial security showed consistent effects across models, particularly on the environmental QoL. These results suggest that overall physical activity significantly mediates the relationship between nature exposure and all examined QoL domains, in contrast to previous models using physical activity in nature, where mediation was limited to the psychological QoL domain. Indirect bootstraped effects from nature exposure to QoL domains were also significant (physical B = 1.31, 95% BootCI = 0.89–1.80, psychological B = 1.43, 95% BootCI = 0.97–1.93, social B = 0.94, 95% BootCI = 0.39–1.55, environmental B = 0.77, 95% BootCI = 0.42–1.14).

Table 5 summarizes all bootstrapped indirect effects (5000 resamples) of nature exposure on the four QoL domains via PA in nature and overall PA. For PA in nature, only the psychological domain showed a significant indirect effect. Indirect effects for physical, social, and environmental QoL were not significant. In contrast, overall PA significantly mediated the associations between nature exposure and all four QoL. Table 5 also reports the corresponding direct and total effects; the proportion mediated is shown only for significant indirect effects.

## 4. Discussion

Exposure to nature and green spaces in one’s living environment may encourage individuals to spend more time outdoors and engage in physical activity—an important contributor to health, well-being, and overall quality of life (Calogiuri & Chroni, 2014; Marquez et al., 2020; Yen et al., 2021). However, although increasing evidence indicates that nature exposure can amplify the positive effects of physical activity, little research has explored how these benefits interact with each other or work in combination (Shanahan et al., 2016). In the present study, we examined whether overall physical activity and physical activity in nature mediate the relationships between nature exposure and the physical, psychological, social, and environmental domains of quality of life. The results supported our hypotheses, showing that overall physical activity serves as a significant mediator in these associations. Our findings align with previous research demonstrating the mediating role of overall physical activity in the relationship between nature exposure and psychological (van den Berg et al., 2019; Kruja et al., 2024; Liao et al., 2023; Murrin et al., 2023; Sugiyama et al., 2008) and physical well-being (Kruja et al., 2024). The present study extends this understanding by providing evidence that overall physical activity mediates associations between nature exposure and social and environmental domains of quality of life. We will discuss these new findings in more detail below.

Greater exposure to nature and living in areas with more greenery may contribute to social cohesion, a sense of community, and feelings of safety by creating vital neighborhood spaces for social interactions (Korpela et al., 2014). Our study provides empirical evidence that nature exposure is positively associated, both directly and indirectly through overall physical activity, with increased satisfaction in personal relationships (i.e., the quality of relationships with family, friends, and significant others) and social support (the availability and adequacy of support from friends, family, and other social networks). These results are consistent with those of previous studies, which suggest that exposure to natural environments can enhance social relationships through physical and recreational activities in nature (Mensah et al., 2016). This highlights the importance of accessible green spaces (parks, community gardens, green corridors) as vital resources for social connection and relationship satisfaction.

Overall physical activity also mediated the associations between nature exposure and the environmental domain of quality of life, suggesting that if people have higher nature exposure, they evaluate their physical environment, living conditions, and resources more positively, such as safety, access to health and social services, opportunities for recreation, and transportation. Previous studies reported that more natural environments in urban regions help to mitigate heat and air pollution and promote biodiversity, contributing to a greater quality of life (Mensah et al., 2016). The findings of the present study suggest that the effect of nature exposure on a more positive evaluation of the living environment is also mediated by physical activity. Specifically, our study shows that how people use these environments (through physical activity) also might shape their subjective evaluations of environmental quality. Physical activity may serve as a mechanism through which individuals engage more actively and meaningfully with their environment, thereby shaping how they perceive and evaluate it. When people regularly move through natural settings—whether walking, cycling, or exercising—they are more likely to experience their surroundings directly, notice their qualities (e.g., cleanliness, safety, accessibility), and develop a stronger sense of place attachment and environmental awareness. Additionally, physical activity often leads individuals to explore a wider range of local amenities—such as parks, walking paths, or community green areas—which can enhance their perception of available resources and services (e.g., recreation opportunities, transportation access, safety). Our findings partially overlap with those of a previous study, which suggested that visits to nature, a connection to nature, and levels of physical activity were related to the adoption of pro-environmental behaviors (Teixeira et al., 2023).

Notably, physical activity in nature only mediated the relationship between nature exposure and the psychological domain of quality of life. One possible explanation of this finding is that physical activity in natural environments has been shown to offer unique psychological benefits beyond those of general physical activity (Laezza et al., 2025; Lahart et al., 2019; Pretty et al., 2005; Wicks et al., 2022). Further, physical activity in natural settings is more directly related to stress reduction, emotional regulation, and mental well-being than overall physical activity (Bratman et al., 2021; Lahart et al., 2019). Previous findings also showed that neighborhood greenness was more strongly associated with mental health than it was with physical health (Sugiyama et al., 2008). Overall physical activity includes a range of behaviors (e.g., active commuting, household chores, indoor exercise, and structured workouts), which can influence multiple domains of quality of life, not just the psychological. Thus, overall physical activity mediated associations across all four quality-of-life domains, whereas nature-based activity mediated only the psychological domain. Finally, in many urban lifestyles, opportunities for physical activity in nature may be less frequent or more limited than other forms of activity (e.g., walking indoors, commuting), indicating that it might have a stronger but more specific influence on mental well-being (Pasanen et al., 2018).

In the present study, we tested associations between nature exposure, physical activity, and quality of life, controlling for financial security. The regression analyses showed that financial security, together with nature exposure, was related to physical activity in nature, and it was also significantly associated with all domains of quality of life when analyzed alongside nature exposure and physical activity in nature. Although financial security was not significantly associated with the overall physical activity, it still contributed—in combination with nature exposure and physical activity in nature—to explaining psychological, physical, social, and environmental domains of quality of life. These results suggest several important insights into the complex relationships between nature exposure, physical activity, financial security, and quality of life. Specifically, people who feel financially secure may be more able to spend time in natural environments, whether through leisure, recreation, or other physical activity. Financial security may provide the time, resources, and perceived safety needed to access and enjoy natural spaces. The fact that financial security, exposure to nature, and physical activity in nature together explain all four domains of quality of life—psychological, physical, social, and environmental—underscores the need for policies that aim to reduce inequality in terms of exposure to nature and nature-based physical activity (Christiana et al., 2021).

Finally, the finding that financial security is associated with physical activity in nature, but not with overall physical activity, suggests that nature-based activity may be less accessible to financially insecure individuals, even if they are physically active in other ways (e.g., through work or chores). This points to a disparity in the quality and context of physical activity—people with lower financial security may not have the time, resources, or safe environments to engage in restorative, leisure-based activity in natural settings. Previous studies also reported that deprived populations often reside in neighborhoods that are farther from green spaces and are unsafe, noisy, and more polluted compared to other areas (European Environment Agency, 2018; Schüle et al., 2019). Efforts to promote health through nature-based activity must address structural barriers—such as lack of nearby green spaces, safety concerns, or time constraints—that disproportionately affect low-income populations (Harrison et al., 2023).

Our findings have several practical implications. First, urban planners should not only increase the amount of green space but design it to actively invite movement—e.g., through walking trails, bike paths, outdoor fitness equipment, or multi-use recreational areas. This can enhance both environmental satisfaction and health outcomes by encouraging direct, positive engagement with the natural environment. Next, in disadvantaged communities with limited access to natural environments, initiatives that simultaneously improve nature exposure and promote physical activity in nature could help strengthen the quality of life.

The present study has several important limitations that should be acknowledged. First, due to its cross-sectional design, the directionality of the observed associations cannot be determined, and the relationships between variables may be bidirectional. While our model assumes that nature exposure promotes physical activity, which in turn enhances quality of life, reverse causality cannot be ruled out. For example, individuals with a higher quality of life may be more likely to engage in physical activity and seek out natural environments, or physically active individuals may preferentially choose outdoor settings, thereby increasing their exposure to nature. Longitudinal studies are needed to disentangle these temporal relationships. Second, the study did not assess the specific type of nature exposure or the characteristics of greenery in participants’ environments. Previous research has shown that different types of green spaces may be associated with different physical activities—for example, urban green areas are linked to more sports and cycling, while exposure to agricultural green spaces is more related to gardening (Picavet et al., 2016). The results of a previous study suggested that only green physical activity for leisure, not other forms such as transportation, mediates the relationship between perceived greenspace and mental well-being (Li et al., 2023). Because our study did not distinguish between types of nature exposure, this may have influenced the findings and introduced potential bias.

Also, nature-based physical activity was assessed using a single retrospective frequency item averaged across four seasons. This makes it vulnerable to recall and social desirability bias and means that duration and intensity data are omitted. It is recommended that future studies use more specific questions. Also, we used different instruments to assess general physical activity and physical activity in nature. Although both measures capture related aspects of physical activity, they were assessed using different instruments and over different seasonal timeframes. This inconsistency is a methodological limitation. Another important limitation is that we only measured overall physical activity in the present study, so it is unclear whether this activity occurred indoors, outdoors, or as a combination of both. It has been concluded that people enjoy outdoor exercise more than indoor physical activity (Peddie et al., 2024), which might have impacted our results.

Next, our sample is not representative, as it consists predominantly of women (74%) and individuals with a high level of education, which limits the generalizability of the findings. The present sample predominantly consists of women (74%) and participants with university-level education, which may limit the generalizability of the results. Previous studies suggest that women tend to report lower physical and mental quality of life (Alwhaibi & Balkhi, 2025; Sialino et al., 2022), while higher educational attainment is generally associated with better overall quality of life, partly through its links to greater income and improved health (Powdthavee et al., 2015). Therefore, our findings may not fully capture patterns observed among men or individuals with lower levels of education, and their generalizability to the broader population may be limited. Finally, the internal consistency of the Nature Exposure Scale in the present study was only marginal, so the findings should be interpreted with caution and tested in other studies.

## 5. Conclusions

The results of our study showed that while nature exposure was independently associated with better QoL, this relationship was partly explained by physical activity. Specifically, overall physical activity mediated the associations between nature exposure and the psychological, physical, social, and environmental domains of quality of life. Physical activity in nature, however, only mediated the psychological domain. In regression analyses, financial security, together with nature exposure, explained physical activity in nature and was also a significant predictor of all quality-of-life domains. While financial security was not significantly associated with overall physical activity, it contributed to explaining the psychological, physical, social, and environmental domains of quality of life in combination with nature exposure and physical activity in nature. These findings highlight the broader role of physical activity in linking nature exposure to quality of life and underscore the importance of supporting active lifestyles in nature to enhance well-being. Strategies that promote equal access to natural environments and opportunities for engaging in physical activity within them should be developed. Furthermore, longitudinal research is needed to confirm the directionality of these associations and to better understand the long-term impacts of nature-based activity on quality of life.

## Figures and Tables

**Table 1:** Sample characteristics (n = 924).

Characteristics		n	%
Gender	men	244	26.4
	women	680	73.6
Age, years (m ± SD) 40.0 ± 12.4			
Education	secondary	105	11.3
	in full-time studies	71	7.7
	undergraduate degree	385	41.7
	postgraduate degree	322	34.8
	other	41	4.5
Family situation	single	170	18.4
	single, but in a committed relationship	159	17.2
	married	534	57.8
	other	61	6.6
Place of residence	capital	143	15.5
	capital suburb	28	3.0
	provincial city with more than 100,000 inhabitants	318	34.4
	provincial city with more than 10,000 inhabitants	185	20.0
	rural area	250	27.1
Ethnicity group	ethnic majority	842	91.1
	ethnic minority	27	2.9
	not sure	55	6.0
Financial security	less secure compared to others	158	17.1
	same	599	64.8
	more secure compared to others	167	18.1
Body mass group	underweight	35	3.8
	normal weight	514	55.7
	overweight	261	28.3
	obesity	112	12.2

Note. m—mean; SD—standard deviation.

**Table 2:** Correlation matrix between the study variables (n = 924).

Variables	1	2	3	4	5	6	7	8
1. Financial security	1.00							
2. Nature exposure	0.15 **	1.00						
3. PA in nature	0.11 **	0.37 **	1.00					
4. Overall PA	0.08 *	0.29 **	0.42 **	1.00				
5. Physical QoL	0.26 **	0.24 **	0.16 **	0.28 **	1.00			
6. Psychological QoL	0.26 **	0.30 **	0.20 **	0.30 **	0.66 **	1.00		
7. Social QoL	0.16 **	0.15 **	0.10 **	0.15 **	0.48 **	0.57 **	1.00	
8. Environmental QoL	0.36 **	0.31 **	0.16 **	0.22 **	0.59 **	0.64 **	0.51 **	1.00
m ± SD	2.01 ± 0.59	4.10 ± 0.73	3.51 ± 1.33	86.76 ± 52.07	72.27 ± 15.82	66.86 ± 16.74	64.18 ± 22.69	69.98 ± 15.56

Note. PA—physical activity, m—mean, SD—standard deviation, QoL—quality of life. * *p* < 0.05, ** *p* < 0.01.

**Table 3:** Mediating effect of physical activity in nature in the association between nature exposure and quality-of-life domains, with financial security as a covariate (n = 924).

**Model 1: Nature Exposure → PA in Nature → Physical QoL**			
Outcome: PA in nature: R^2^ = 0.14, F = 74.0, *p* < 0.001 (path a)	B	β	*p*
Nature exposure	0.65	0.35	<0.001
Financial security	0.14	0.06	0.038
Outcome: Physical QoL R^2^ = 0.12 F = 42.7, *p* < 0.001 (paths b and c’)			
Nature exposure	3.88	0.18	<0.001
Financial security	6.51	0.24	<0.001
PA in nature	0.81	0.07	0.042
**Model 2: Nature exposure → PA in nature → psychological QoL**			
Outcome: Psychological QoL R^2^ = 0.15, F = 53.1, *p* < 0.001 (paths b and c’)			
Nature exposure	5.34	0.23	<0.001
Financial security	6.35	0.23	<0.001
PA in nature	1.09	0.09	0.008
**Model 3: Nature exposure → PA in nature → social QoL**			
Outcome: Social QoL: R^2^ = 0.05, F = 14.8, *p* < 0.001 (paths b and c’)			
Nature exposure	3.49	0.11	0.001
Financial security	5.63	0.15	<0.001
PA in nature	0.71	0.04	0.227
**Model 4: Nature exposure → PA in nature → environmental QoL**			
Outcome: Environmental QoL: R^2^ = 0.20, F = 79.0, *p* < 0.001 (paths b and c’)			
Nature exposure	5.21	0.24	<0.001
Financial security	8.78	0.33	<0.001
PA in nature	0.33	0.03	0.368

Note. B—unstandardized, β—standardized regression coefficient; PA—physical activity, QoL—quality of life; a—effect from the independent variable to the mediator, b—effect from the mediator on the outcome, c’—direct effect from the independent variable on the outcome.

**Table 4:** Mediating effect of overall physical activity in the association between nature exposure and quality-of-life domains, with financial security as a covariate (n = 924).

**Model 1: Nature Exposure → Overall PA → Physical QoL**			
Outcome: Overall PA: R^2^ = 0.09, F = 43.3, *p* < 0.001 (path a)	B	β	*p*
Nature exposure	20.26	0.28	<0.001
Financial security	3.89	0.04	0.164
Outcome: Physical QoL R^2^ = 0.16, F = 58.4, *p* < 0.001 (paths b and c’)			
Nature exposure	3.10	0.14	<0.001
Financial security	6.37	0.24	<0.001
Overall PA	0.06	0.21	<0.001
**Model 2: Nature exposure → overall PA → psychological QoL (paths b and c’)**			
Outcome: Psychological QoL R^2^ = 0.19, F = 69.7, *p* < 0.001			
Nature exposure	4.62	0.20	<0.001
Financial security	6.24	0.22	<0.001
Overall PA	0.07	0.22	<0.001
**Model 3: Nature exposure → overall PA → social QoL (paths b and c’)**			
Outcome: Social QoL: R^2^ = 0.05, F = 17.8, *p* < 0.001			
Nature exposure	3.01	0.10	0.004
Financial security	5.56	0.15	<0.001
Overall PA	0.05	0.11	0.002
**Model 4: Nature exposure → overall PA → environmental QoL (paths b and c’)**			
Outcome: Environmental QoL: R^2^ = 0.22, F = 85.9, *p* < 0.001			
Nature exposure	4.67	0.22	<0.001
Financial security	8.68	0.33	<0.001
Overall PA	0.04	0.13	<0.001

Note. B—unstandardized, β—standardized regression coefficient; PA—physical activity, QoL—quality of life; a—effect from the independent variable to the mediator, b—effect from the mediator on the outcome, c’—direct effect from the independent variable on the outcome.

**Table 5:** Bootstrapped indirect effects of nature exposure on the four domains of quality of life (QoL) mediated by physical activity in nature and overall physical activity (n = 924).

Mediator	QoL Domain	Indirect Effect (B)	95% BootCI	Sig.	Direct Effect c′ (B)	Total Effect c (c′ + Indirect)	% Mediated †
PA in nature	Physical	0.53	−0.01–1.08	ns	3.88	4.41	—
	Psychological	0.71	0.15–1.29	*	5.34	6.05	11.7%
	Social	0.47	−0.31–1.29	ns	3.49	3.96	—
	Environmental	0.22	−0.28–0.75	ns	5.21	5.43	—
Overall PA	Physical	1.31	0.88–1.81	**	3.09	4.40	29.8%
	Psychological	1.43	0.98–1.96	**	4.62	6.05	23.6%
	Social	0.94	0.40–1.58	**	3.01	3.95	23.8%
	Environmental	0.77	0.41–1.15	**	4.67	5.44	14.2%

Note. PA—physical activity, QoL—quality of life, B—unstandardized regression coefficient, BootCI—bootstrapped confidence interval, Sig.—statistical significance; † proportion mediated − (indirect/total) × 100, reported only when the indirect effect was significant (BootCI excluding 0); ns—non-significant, * *p* < 0.05, ** *p* < 0.01.

## Data Availability

The dataset generated and analysed during the current study is not publicly available, but is available from the corresponding author upon reasonable request.
